# Activation of Mir-29a in Activated Hepatic Stellate Cells Modulates Its Profibrogenic Phenotype through Inhibition of Histone Deacetylases 4

**DOI:** 10.1371/journal.pone.0136453

**Published:** 2015-08-25

**Authors:** Ying-Hsien Huang, Mao-Meng Tiao, Li-Tung Huang, Jiin-Haur Chuang, Kuang-Che Kuo, Ya-Ling Yang, Feng-Sheng Wang

**Affiliations:** 1 Departments of Pediatrics, Kaohsiung Chang Gung Memorial Hospital and Chang Gung University College of Medicine, Kaohsiung, Taiwan; 2 Departments of Surgery, Kaohsiung Chang Gung Memorial Hospital and Chang Gung University College of Medicine, Kaohsiung, Taiwan; 3 Departments of Anesthesiology, Kaohsiung Chang Gung Memorial Hospital and Chang Gung University College of Medicine, Kaohsiung, Taiwan; 4 Departments of Medical Research, Kaohsiung Chang Gung Memorial Hospital and Chang Gung University College of Medicine, Kaohsiung, Taiwan; Vrije Universiteit Brussel, BELGIUM

## Abstract

**Background:**

Recent studies have shown that microRNA-29 (miR-29) is significantly decreased in liver fibrosis and that its downregulation influences the activation of hepatic stellate cells (HSCs). In addition, inhibition of the activity of histone deacetylases 4 (HDAC4) has been shown to strongly reduce HSC activation in the context of liver fibrosis.

**Objectives:**

In this study, we examined whether miR-29a was involved in the regulation of HDAC4 and modulation of the profibrogenic phenotype in HSCs.

**Methods:**

We employed miR-29a transgenic mice (miR-29aTg mice) and wild-type littermates to clarify the role of miR-29a in cholestatic liver fibrosis, using the bile duct-ligation (BDL) mouse model. Primary HSCs from both mice were treated with a miR-29a mimic and antisense inhibitor in order to analyze changes in profibrogenic gene expression and HSC activation using real-time quantitative RT-PCR, immunofluorescence staining, western blotting, and cell proliferation and migration assays.

**Results:**

After BDL, overexpression of miR-29a decreased collagen-1α1, HDAC4 and activated HSC markers of glial fibrillary acidic protein expression in miR-29aTg mice compared to wild-type littermates. Overexpression of miR-29a and HDAC4 RNA-interference decreased the expression of fibrotic genes, HDAC4 signaling, and HSC migration and proliferation. In contrast, knockdown of miR-29a with an antisense inhibitor increased HDAC4 function, restored HSC migration, and accelerated HSC proliferation.

**Conclusions:**

Our results indicate that miR-29a ameliorates cholestatic liver fibrosis after BDL, at least partially, by modulating the profibrogenic phenotype of HSCs through inhibition of HDAC4 function.

## Introduction

Persistent liver injury due to cholestasis and hepatitis may result in liver fibrosis that engages a range of cell types [1, 2]. Liver fibrosis is a complex process modulated by a set of signaling pathways. Following acute or chronic liver injury of any etiology, hepatic stellate cells (HSCs) are activated and undergo morphologic and functional trans-differentiation, transforming from vitamin A-storing cells into contractile myofibroblastic cells responsible for extracellular matrix (ECM) production in the injured liver [1–3]. It is well known that the stimulation of HSCs by transforming growth factor-β (TGF-β) is a crucial event in liver fibrogenesis because of its impact on myofibroblast transition and ECM induction.

MicroRNAs (miRs) are single-stranded 21–22 nucleotide non-coding RNAs that are capable of controlling gene expression at the post-transcriptional level by silencing endogenous mRNA transcripts in a process referred to as RNA interference (RNAi) [4]. Recent studies have shown that the expression of miR-132 and miR-29, which consists of miR-29a, miR-29b, and miR-29c, are significantly decreased in fibrotic livers, as demonstrated in human liver cirrhosis as well as in two different models of liver injury induced by bile duct ligation (BDL) and carbon tetrachloride (CCl_4_) [5]. *In vitro* activation of HSCs led to a downregulation of all miR-29-members during eight days of culturing [5]. Moreover, overexpression of miR-29 in murine HSCs resulted in a downregulation of collagen expression through directly targeting the mRNA expression of ECM genes. [5, 6] In contrast, another study reported increased fibrosis and mortality in miR29ab1-knockout mice following the administration of CCl_4_ [7]. Serum levels of miR-29a are significantly lower in patients with advanced liver cirrhosis than in healthy controls or patients with early fibrosis [5]. Because liver fibrosis is an imbalance between ECM deposition and ECM degradation, the miR-29-mediated suppression of ECM synthesis in HSCs could hopefully drive the balance toward reduced fibrosis.

Histone deacetylase (HDAC) 4, a member of the class II HDACs, has been found to modify acetylation reactions in histones and non-histone proteins, and has been reported to regulate diabetes-induced fibrosis [8], idiopathic pulmonary fibrosis [9] and liver fibrosis [10]. Administration of HDAC inhibitors ameliorates both in experimental liver and kidney fibrosis [11]. In addition, inhibition of HDAC activity leads to a strong reduction of HSC activation through the induction of miR-29 expression [10]. Moreover, our group has demonstrated that HDAC4 interference increases the acetylation status of H3K9, which is enriched in the miR-29a proximal promoter [12]. In addition, our group also demonstrated that miR-29a signaling protects against glucocorticoid-induced osteoporosis and hyperglycemia-induced renal fibrosis through a reduction in HDAC4 signaling [12, 13]. Indeed, bioinformatic searches indicate that HDAC4 are predicated to be putative miR-29a targets (http://microrna.sanger.ac.uk and www.microrna.org). Moreover, we have previously demonstrated that overexpression of miR-29a significantly reduces the expression of pro-apoptotic proteins and enhances the expression of phospho-AKT proteins, resulting in a decrease in cellular apoptosis, liver injury, and fibrosis in cholestasis [14]. We proposed that miR-29a interacted with HDAC signaling to regulate HSC activation in liver fibrosis. In this study, we employed miR-29a transgenic mice (miR-29aTg mice) to clarify the role of miR-29a in hepatic injury and fibrogenesis in an experimental BDL liver fibrosis model.

## Materials and Methods

### Ethics statement

Our animal protocol was reviewed and approved by the Institutional Animal Care and Use Committee (IACUC) of the Chang Gung Memorial Hospital (#2012090301). FVB male mice (National Animal Center of Academia Sinica, Taipei, Taiwan) weighing 25–35 g were purchased from BioLASCO Taiwan Co., Ltd. All animals were housed in an animal facility at 22°C, with a relative humidity of 55%, in a 12 h light/12 h dark cycle, with food and sterile tap water available *ad libitum*.

### Construction and breeding of miR-29a transgenic mouse colony

The PGK promoter and miR-29a precursor fulllength sequence were cloned from the cDNA library by PCR protocols. The cDNAs were then inserted into the pUSE empty expression vector; and the linear human PGK-miR-29a-BGH poly-A cDNAs were cloned. The designed constructs were transferred into fertilized eggs from FVB/N mice (BioLASCO Taiwan Co., Ltd). The eggs were further transferred into Crl: CD1 foster mothers, as previously described [15]. Transgenic mice were bred in a specific pathogen-free condition and genotyped by PCR using specific primers (forward: 5’-GAGGATCCCCTCAAGGATACCAAG- GGATGAAT-3’ and reverse 5’-CTTCTAGAAGGAGTGTTTCTAGGTATCCGT- CA-3’) [12].

### Animal model and experimental protocol

FVB male mice were used for all of the experiments. The mice were randomly divided into either the “BDL” group or the “sham” group, depending on whether the mice had received a ligation or a sham ligation of the common bile duct, as described in a previous study [14]. The mice were sacrificed one week after the procedure. Liver tissues were snap-frozen so that mRNA and protein expression could be determined later. The samples were kept at −80°C prior to biochemical analysis.

### Primary HSC isolation and culture

Primary HSCs were isolated from livers of miR-29aTg mice or WT littermate by sequential digestion of the liver with pronase and collagenase, followed by density gradient centrifugation in 8.5% Nycodenz (Sigma-Aldrich, St. Louis, MO) as described previously [16, 17]. The purity of the HSCs was assessed by autofluorescence of stored retinoids in HSC lipid droplets (S1 Fig). Cell viability determined by a Trypan Blue exclusion assay revealed that more than 95% of the cells were viable. Purity of the HSC culture was found to be 95%–99% by oil red O staining [17]. Cells were maintained in Dulbecco’s modified Eagle’s medium supplemented with 5% newborn calf serum. After 1 day in culture, the HSCs had a quiescent phenotype and they developed an activated phenotype after 7–14 days. The passage of the cultured cells was conducted after reaching confluence and experiments were carried out using cells between passages 2 and 6.We compared cell survival, profibrogenic gene expression, cell proliferation and migration in primary activated HSCs from wild-type (WT) and miR-29aTg mice. Thereafter, miR-29a or HDAC4 RNAi was added to the activated HSC culture. To investigate the mechanism why cholestasis may affect HSC activation, we treated primary HSCs with one of the hydrophobic bile acids, taurolithocholic acid (TLCA) (Sigma) for 24 hours. Four independent *in vitro* experiments were performed.

### RNAi transfection

HSCs were transfected with a miR-29a precursor (a miR-29a mimic, C-300504, Lafayette, USA), miR-29a antisense oligonucleotide inhibitor (IH-300504, Lafayette, USA), or miR control (N-00100 Thermo, Lafayette, USA) for 24 hours. Cells were seeded into plates (5 × 10^5^ cells/well, 6-well plate for western blotting and real-time quantitative RT-PCR; 10^4^ cells/well, 12-well plate for immunofluorescence), incubated overnight and transfected using DharmaFECT siRNA Transfection Reagent (Lafayette, USA) as instructed [18]. HDAC4 RNAi (sc-35541, Santa Cruz, USA), which targets HDAC4, were transfected into primary HSCs for 24 hours using the Lipofectamine RNAiMAX Transfection Reagent (Invitrogen, Carlsbad, CA), according to the manufacturer's instructions [19].

### RNA isolation and real-time quantitative RT-PCR

In order to quantify miR-29 in the tissue samples, we performed real-time, quantitative RT-PCR with the ABI 7700 Sequence Detection System (TaqMan; Applied Biosystems, Inc., Foster City, CA). Total miR was isolated using the MicroRNA Isolation kit (BioChain Institute, Inc, Hayward, CA), according to the manufacturer’s instructions. U6 gene (Applied Biosystems, Foster City, CA) expression was used to normalize gene and miR expression. Templates were pre-amplified using 2× TaqMan PreAmp Master Mix and 10X Megaplex PreAmp Primers, and then PCR-amplified using 2× TaqMan Universal PCR Master Mix. Relative quantification of gene expression was based on the comparative threshold cycle (C_T_) method in which the amount of the target was determined to be 2^-(ΔCT target – Δ CT calibrator)^ or 2^-ΔΔCT^. PCR products were then electrophoresed on a 2% agarose gel in order to confirm the amount of the products. Primers were designed to amplify collagen-1α1 (forward, 5′-ACCCTGGAAACAGACGA-3′; reverse, 5′-TTTGGTAAGGTTGAATGCACT-3′), collagen-3α1 (forward, 5′-TACCTCAACTGGTCAGAACAGATA-3′; reverse, 5′-GTACTCCTTCAAATTCCTGCT-3′), monocyte chemoattractant protein-1 (MCP-1) (forward, 5′-TTGACCCGTAAATCTGAAGCTA-3′; reverse, 5′-ATTAAGGCATCACAGTCCG-3′) and GAPDH (forward, 5′-CACTGCCACCCAGAAGA-3′; reverse, 5′-TCCACGACGGACACATT-3′). Validation experiments were performed in duplicate, and amplification efficiencies were validated.

### Immunofluorescence

Liver tissues were embedded in TissueTek optimal cutting temperature (OCTTM) compound (Sakura Finetek) and frozen at −80°C for storage. Frozen sections (4 μm thick) were prepared using a cryostat (CM3050 S, Leica) and processed for Sirius Red staining. Cryosections were fixed with an isotonic PBS and 4% paraformaldehyde solution for 1 h. To block non-specific background staining, the samples were incubated in a solution containing 1% BSA for 30 min. After washing with PBS, the slides were incubated with the primary antibodies. Anti-glial fibrillary acidic protein (GFAP) (ab10062, abcam), anti-HDAC4 (#5392, cell signaling, MA), and anti-a-SMA (ab5694, abcam, UK) primary antibodies were used. Fluor 488-conjugated (green) and Alexa Fluor 595 (red)-conjugated secondary antibodies (Molecular Probes) were used. Samples were co-stained with 4ʹ,6-diamidino-2-phenylindole (DAPI; Molecular Probes) to facilitate visualization of the nuclei. The stained cells were mounted with a fluorescent mounting medium (Dako Cytomation) and visualized by microscopy (Olympus). The exposure gains and rates were consistent between samples. Fluorescent intensities were quantified on independent color channels by using Image J analysis.

### Western blot analysis

Extraction of cytoplasmic and nuclear fractions was performed using NE-PER Nuclear and Cytoplasmic Extraction Reagents Kit (Pierce, Rockford, IL, USA) according to the manufacturer’s protocol. Crude proteins (30 μg) were treated with sample buffer, boiled for 10 min, separated using 8–15% sodium dodecyl sulfate–polyacrylamide gels, and transferred to a nitrocellulose membrane. Blots were incubated with the primary antibodies against collagen-1α1 (#sc-8784-R, Santa Cruz Biotechnology, Dallas, TX), HDAC4 (#5392s, Cell Signaling, MA), H3K9Ac (ab4441, abcam, UK), and GAPDH (GTX100118, GeneTex, SA), phosph-Smad3 (p-Smad3; ab51451, abcam, UK), Smad 3 (ab40854, abcam, UK), and GAPDH for cytoplasmic protein control, and lamin B (ab16048, abcam, UK) for nuclear protein control. After washing with TBST and incubating with horseradish peroxidase-coupled anti-rabbit immunoglobulin-G antibodies (dilution, 1:10,000) at room temperature for 2 h, the blots were developed with enhanced chemiluminescence detection (GE Healthcare Biosciences AB, Uppsala, Sweden) and exposed to film. The signals were quantified with densitometry.

### Detection of cellular migration using a wound-healing assay

Cells were seeded into ibidi culture-inserts (ibidi GmbH, Martinsried, Germany) at a concentration of 10,000 cells per well. After allowing cells to attach overnight, the culture-insert was gently removed using sterile forceps. Cells were incubated with scramble, miR-29a mimic, miR-29a anti-sense inhibitor or HDAC4 RNAi. Images were taken at 0, 6, and 21 h, and superimposed using PhotoImpact (Adobe). The number of cells that migrated into the wound space were manually counted in three fields per well under a light microscope at 50× magnification. Areas were quantified by image analysis using Image J analysis.

### Cellular proliferation

HSCs were seeded into 12-well plates at a concentration of 5,000 cells/ml. After one week of culture, cells were rinsed with PBS, fixed in methanol, and stained with 200 μl crystal violet. Cells were rinsed with distilled water and air dried. Once dry, cells were lysed with 2% (w/v) sodium deoxycholate solution with gentle agitation. Then, the plates were washed with distilled water at least three times prior to solubilizing the cell layer with 50 μl of 10% glacial acetic acid. Absorbance was measured at 540 nm on a microplate reader (HIDEX Sense Microplate Reader, Turku, Finland).

### Statistical analysis

All values in the figures and tables were expressed as mean ± standard error. Quantitative data were analyzed using the one-way analysis of variance [20] when appropriate. The least significant difference (LSD) test was used for post-hoc testing when appropriate. Correlations between quantitative variables were assessed using Pearson’s coefficient. Two-sided p values less than 0.05 were considered statistically significant.

## Results

### Over-expression of miR-29a significantly reduced liver fibrosis, HDAC4 expression and HSC activation in cholestatic mice

**Fig 1:**
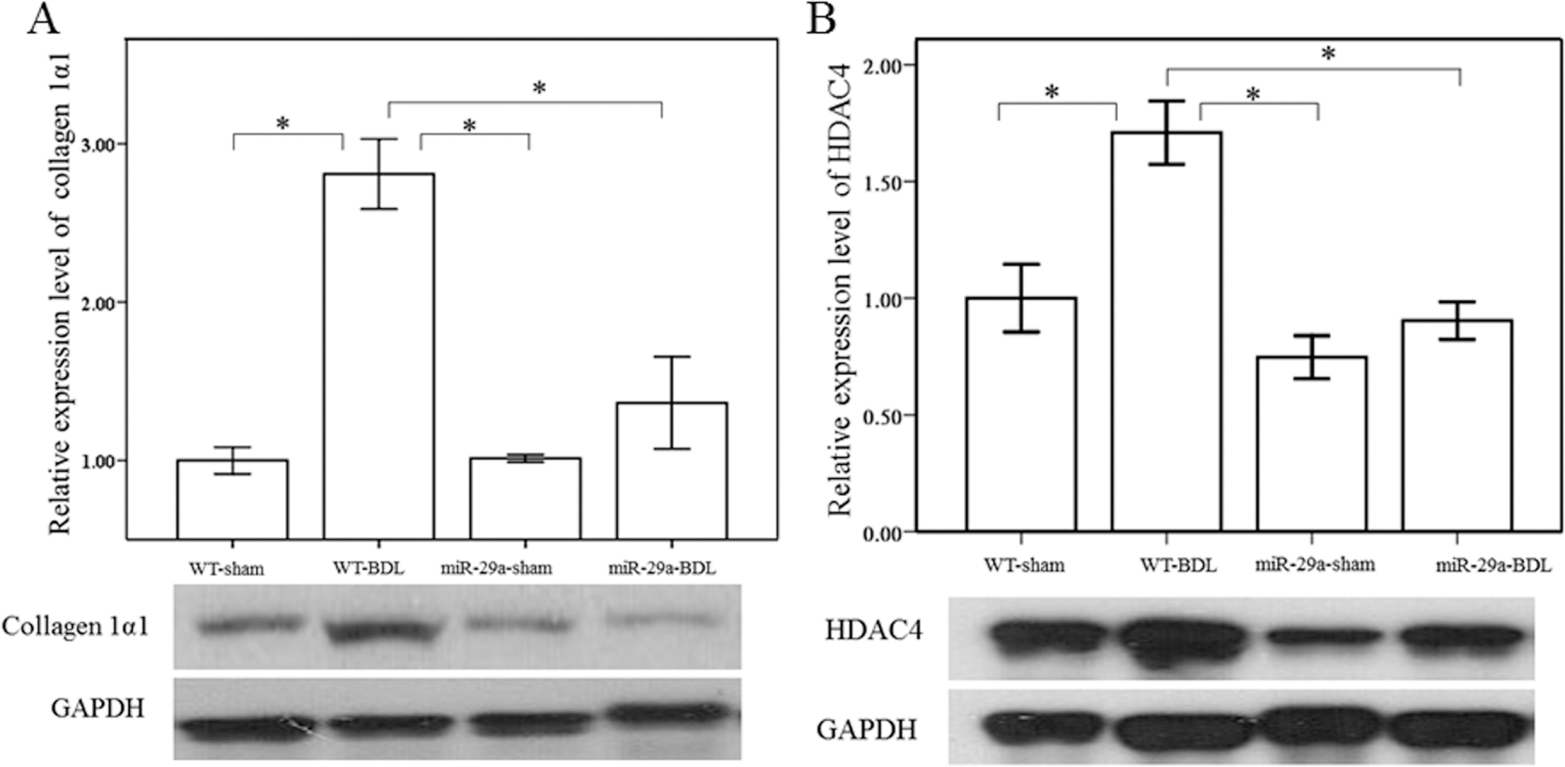
Overexpression of miR-29a in the murine model resulted in downregulation of fibrosis and HDAC4 in the liver of mice after BDL. (A) Sirius-Red staining showed moderate fibrosis in WT mice and mild fibrosis in miR-29aTg mice, which was limited to the close vicinity of the portal area. (B) There was a significantly greater expression of collagen-1α1 and HDAC4 in tissues from the BDL group than in tissues from the sham-operated group in WT mice. Moreover, miR-29a overexpression significantly downregulated collagen-1α1 and HDAC4 protein expression in miR-29aTg mice with cholestasis compared with the WT littermates. Data are expressed as the mean ± SE of six samples per group. *indicates a p < 0.05 between the groups.

To investigate the effect of miR-29a overexpression on the progression of fibrosis, we studied the expression of ECM proteins during hepatic fibrogenesis. As showed in Fig 1A, Sirius-Red staining showed moderate fibrosis in WT mice and mild fibrosis in miR-29aTg mice, which was limited to the close vicinity of the portal area. The relatively moderate fibrosis observed in both mice is due to the Hc-/- genotype (encoding complement factor C5) of the FBV mouse strain used that typically yields less fibrosis when compared to Hc+/+ strains such as BALB/c and C57BL/6J [21]. Therefore, we have performed western blot analysis, we found a significantly higher expression of the collagen-1α1 protein in tissues from the BDL group than in tissues from the sham group (p < 0.001; Fig 1B) in WT mice. However, we observed a weaker induction of collagen-1α1 in miR-29aTg mice with cholestasis (p = 0.188). Moreover, miR-29a overexpression significantly downregulated collagen-1α1 protein expression in miR-29aTg mice with cholestasis compared with the WT littermates (p < 0.001). Then we further characterized HDAC4 protein expression in the liver. As illustrated in Fig 1B, compared with the sham-operation group, the BDL group of WT mice exhibited an increase in HDAC4 protein expression (p = 0.001). Moreover, miR-29a overexpression significantly downregulated HDAC4 immunoreactivity in miR-29aTg mice with cholestasis compared with the WT littermates (p < 0.001), indicating that miR-29a might have an impact on HDAC4 expression in early cholestasis.

**Fig 2:**
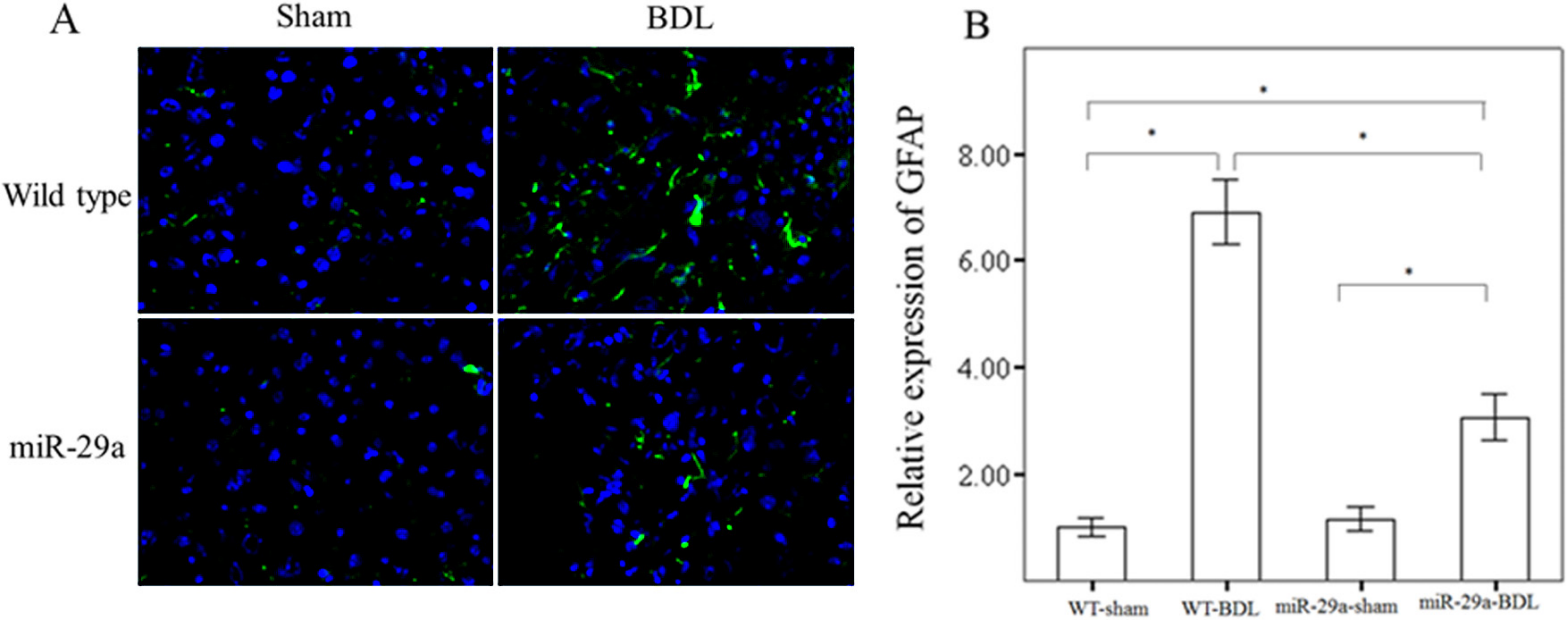
Overexpression of miR-29a decreased GFAP, a marker of HSC, and increased expression in the acute response to injury and immunoreactivity in cholestasis. There was significantly higher expression of GFAP (green) in tissues from the BDL group than in tissues from the sham-operated group in WT mice. Moreover, miR-29a overexpression significantly downregulated GFAP protein expression in miR-29aTg mice with cholestasis compared with the WT littermates. Data are expressed as the mean ± SE of six samples per group. *indicates a p < 0.05 between the groups.

GFAP is a type III intermediate filament protein originally identified in HSC-derived myofibroblasts [22]. It increases in the acute response to injury and decreases in the chronic response [23–25]; and GFAP has therefore been suggested as an early marker of HSC activation. Hence, GFAP expression levels reveal the overall number of HSC and can therefore be utilized as a specific HSC proliferation marker. Compared with the sham-operation group, the BDL group of WT and miR-29aTg mice had increased GFAP protein expression (p < 0.001 and p = 0.003, respectively).(Fig 2) Moreover, miR-29a overexpression significantly downregulated GFAP immunoreactivity in the miR-29aTg mice with cholestasis compared with the WT littermates (p < 0.001).

### Overexpression of miR-29a significantly reduced expression of profibrogenic genes and HDAC4 in activated HSCs

**Fig 3:**
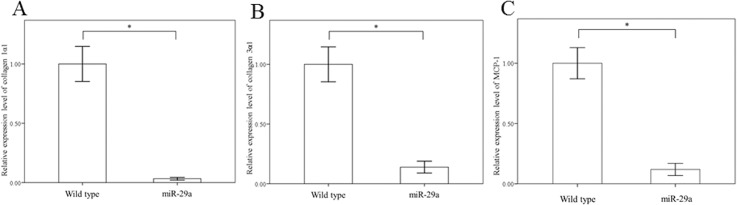
Comparison of collagen-1α1 (A), collagen-3α1 (B) and MCP-1 (C) expression in activated HSCs of WT and miR-29Tg mice. Data are expressed as the mean ± SE of four independent experiments. *indicates a p < 0.05 between the groups.

**Fig 4:**
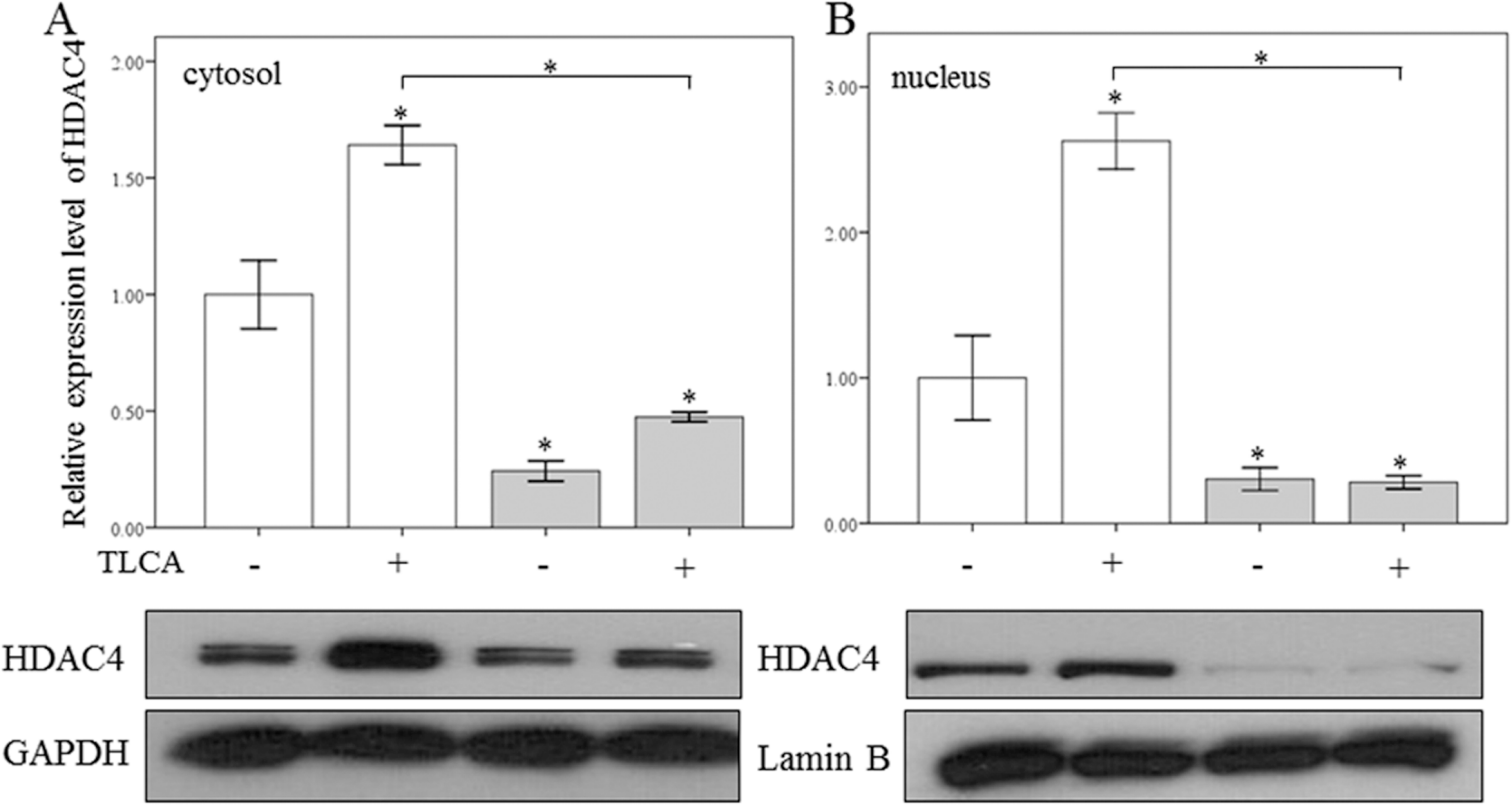
Effects of TLCA on HDAC4 expression in cultured primary HSCs. TLCA increased HDAC4 expression (A) and nuclear translocation (B) in HSCs of WT mice (white bar). HSCs of miR-29a transgenic mice (gray bar) exhibited significantly reduced upregulation and nuclear translation of HDAC4 in response to TLCA stimulation. Data are expressed as the mean ± SE of four independent experiments. *indicates a p < 0.05 between the groups.

The activation of HSCs is known to result in increased expression of several profibrogenic genes, including collagen-1α1, collagen-3α1 and MCP-1. As shown in Fig 3, miR-29a overexpression significantly downregulated the expression of collagen-1α1, collagen-3α1, and MCP-1 in activated HSCs of miR-29aTg mice compared with WT littermates (all p < 0.001). We then treated primary HSCs with one of the hydrophobic bile acids, taurolithocholic acid (TLCA; Sigma), to explore whether miR-29a affects HDAC4 expression and nuclear translocation in response to cholestasis. As shown in Fig 4, we found that there was significant upregulation and nuclear translation of HDAC4 in the HSCs of WT mice following TLCA stimulation (p = 0.001 and p < 0.001, respectively). HSCs of miR-29aTg mice could significantly reduce the upregulation and nuclear translation of HDAC4 in response to TLCA stimulation in HSCs (p = 0.003 and p < 0.001, respectively).

### miR-29a regulates HDAC4 expression and histone acetylation in primary HSCs

**Fig 5:**
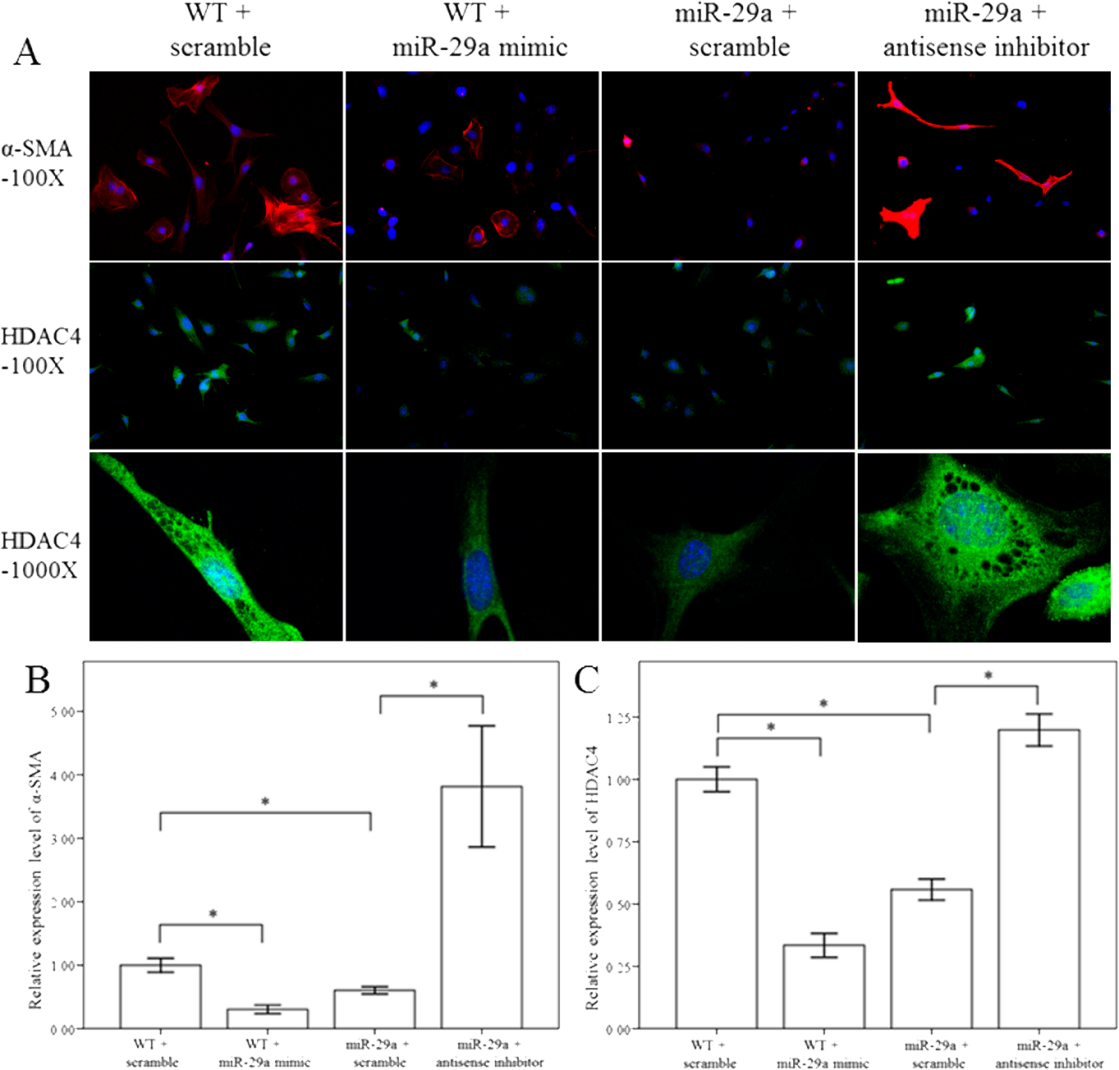
Comparison of α-SMA and HDAC4 expression in 10th culture day of activated HSCs of WT and miR-29aTg mice after treatment with a miR-29a mimic and anti-sense inhibitor for 24 hours. Expression of α-SMA (red) and HDAC4 (green) were much greater in activated HSCs of WT mice than in miR-29aTg mice. Treatment with a miR-29a mimic in activated HSCs of WT inhibited α-SMA and HDAC4 expression as well as HDAC nuclear translocation. In contrast, treatment with miR-29a anti-sense inhibitor in miR-29aTg mice increased α-SMA and HDAC4 expression as well as HDAC4 nuclear translocation. Data are expressed as the mean ± SE of four independent experiments. *indicates a p < 0.05 between the groups.

**Fig 6:**
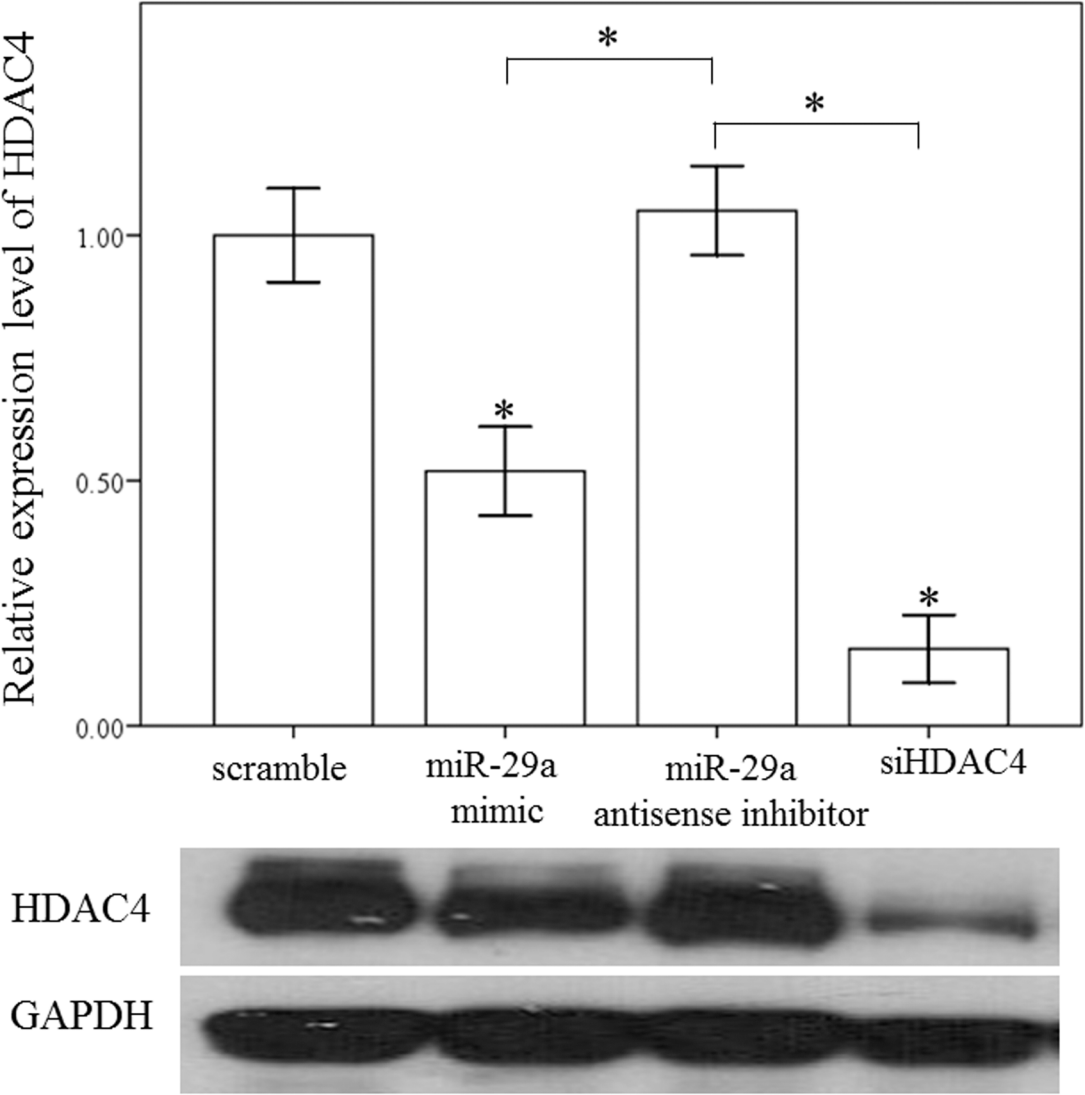
Overexpression of miR-29a decreased HDAC4 expression in primary HSCs. Treatment with a miR-29a mimic and HDAC4 RNAi significantly deceased HDAC4 expression in the HSCs of WT mice. Data are expressed as the mean ± SE of four independent experiments. *indicates a p < 0.05 between the groups.

**Fig 7:**
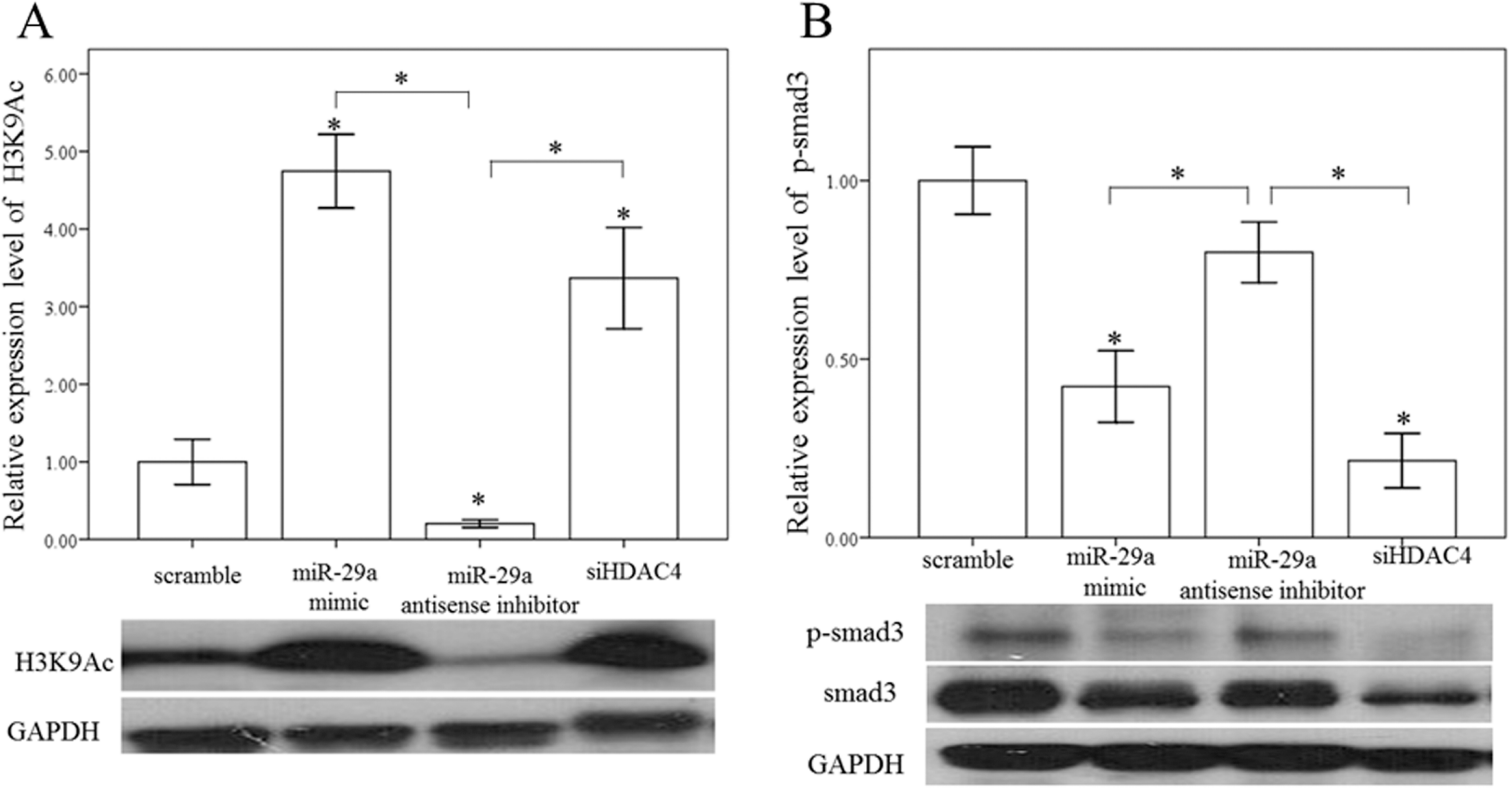
Overexpression of miR-29a increased histone H3 at lysine 9 (H3K9Ac) and decreased Smad3 expression in HSCs. A miR-29a mimic and HDAC4 RNAi significantly increased H3K9Ac and decreased both Smad3 and p-Smad3 expression in HSCs of WT mice. Data are expressed as the mean ± SE of four independent experiments. *indicates a p < 0.05 between the groups.

To test the effects of miR-29a inhibition on the expression of HDAC4, primary HSCs stably expressing a miR-29a mimic, antisense inhibitor, HDAC4 RNAi and scramble control were used. As expected, overexpression of miR-29a significantly downregulated the expression of α-SMA- and HDAC4- in miR-29aTg mice compared than in WT mice (Fig 5A and 5B). In contrast, after treatment with a miR-29a anti-sense inhibitor, α-SMA and HDAC4 expression was significantly upregulated in the HSCs of miR-29aTg mice. Western blotting confirmed the immunofluorescence findings (Fig 6). We then tested whether HDAC4 affected histone acetylation in activated HSCs. Our group has previously demonstrated that HDAC4 interference increased the acetylation status of histone H3 at lysine 9 (H3K9Ac), and observed an enrichment of H3K9Ac in the miR-29a proximal promoter in a diabetic nephropathy animal model [12]. Compared to controls, a miR-29a mimic or HDAC4 RNAi significantly increased the expression of H3K9Ac (p < 0.001 and p = 0.003, respectively; Fig 7). In contrast, addition of a miR-29a anti-sense inhibitor significantly decreased H3K9Ac expression (p = 0.02; Fig 7A). Recent reports have demonstrated that miR-29 acts as a downstream inhibitor and therapeutic miR for TGF-β1/Smad3- mediated renal fibrosis [26] and myogenic differentiation [27]. As shown in Fig 7B, a miR-29a mimic or HDAC4 RNAi inhibited both expression of both Smad3 and p-Smad3 in activated HSCs.

### Gain of miR-29a function inhibits HSC migration and proliferation

**Fig 8:**
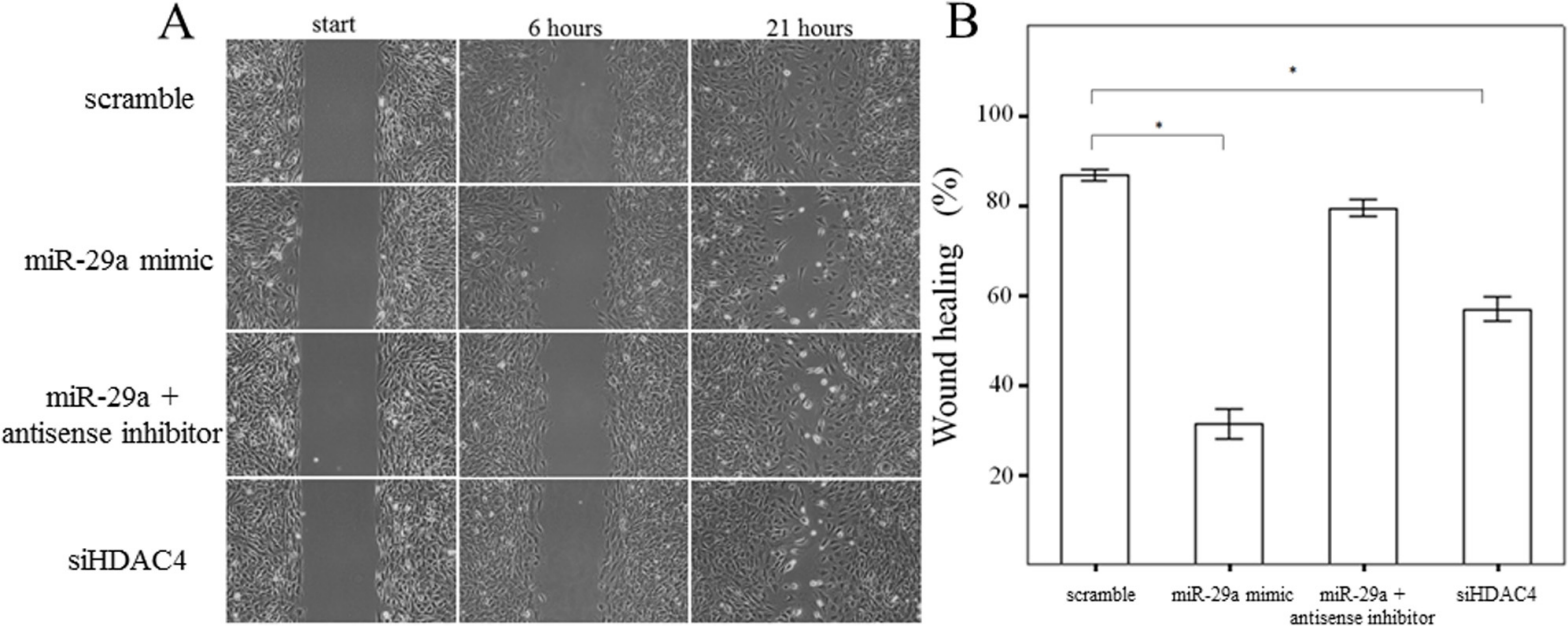
Migration of primary activated HSCs was measured using a wound healing assay. A miR-29a mimic or HDAC4 RNAi significantly inhibited the migration of primary HSCs of WT mice. Data are expressed as the mean ± SE of four independent experiments. *indicates a p < 0.05 between the groups.

**Fig 9:**
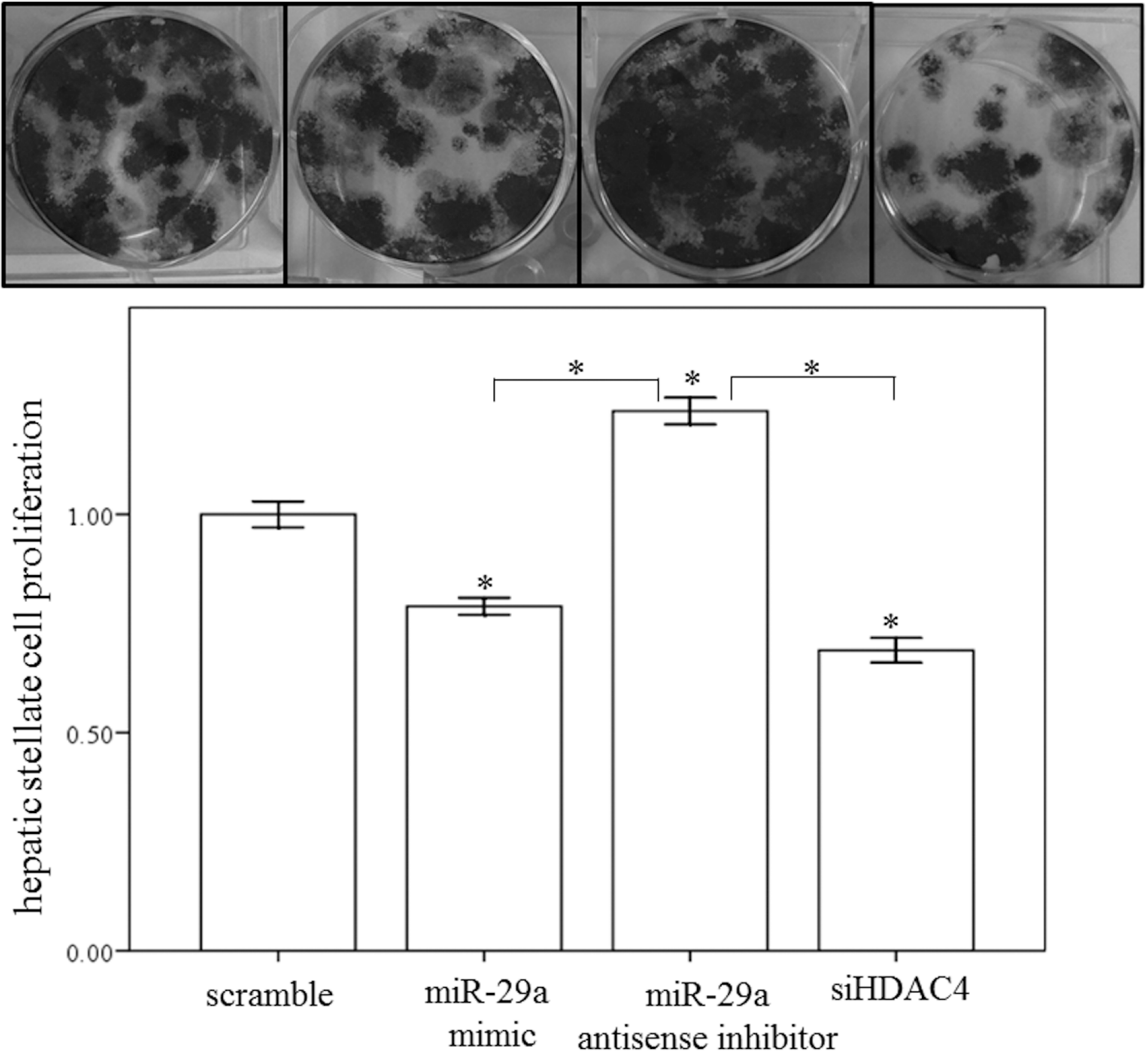
Proliferation of primary activated HSCs was measured by crystal violet assay. A miR-29a mimic or HDAC4 RNAi inhibited the proliferation of primary HSCs. In addition, treatment with a miR-29a anti-sense inhibitor significantly increased HSC proliferation. Data are expressed as the mean ± SE of four independent experiments. *indicates a p < 0.05 between the groups.

In order to assess whether miR-29a may regulate HSC migration, a wound-healing assay was performed using primary HSCs. The results of the present study showed that a miR-29a mimic or HDAC4 RNAi inhibited the migration of primary HSCs (both p < 0.001; Fig 8). We then conducted a cell proliferation assay to examine the effects of miR-29a on cell proliferation. As showed in Fig 9, a miR-29a mimic or HDAC4 RNAi inhibited the proliferation of primary HSCs (both p < 0.001). In addition, addition of a miR-29a anti-sense inhibitor significantly increased HSC proliferation (p < 0.001).

## Discussion

HSC activation and trans-differentiation responsible for ECM production are important features in the pathogenesis of liver fibrosis. To the best of our knowledge, this is the first study to report that miR-29a could directly modulate the profibrogenic phenotype of HSCs in a mouse model of obstructive jaundice in miR-29aTg mice. This study marks the first attempt to shed light on the interplay between miR-29a and HDAC4 signaling upon the activation of HSCs. The main novel findings of this study are as follows. (1) Overexpression of miR-29a in cholestatic mice significantly inhibited liver fibrosis. (2) It also induced a significant decrease in GFAP expression, a type III intermediate filament protein that is expressed in activated HSCs [22]. (3) There was significant upregulation and nuclear translation of HDAC4 in HSCs of WT mice in response to stimulation by the hydrophobic bile acid TLCA. Interestingly, HSCs of miR-29aTg mice significantly reduced the upregulation and nuclear translation of HDAC4 in response to TLCA stimulation. (4) We also observed that overexpression of miR-29a and HDAC4 RNAi significantly downregulated HDAC4, p-Smad3, and the acetylation of histone H3 at lysine 9 (H3K9Ac), which is enriched in the miR-29a proximal promoter [12]. (5) Furthermore, miR-29a overexpression significantly downregulated expression of collagen-1α1, collagen-3α1, and MCP-1 in activated the HSCs of miR-29aTg mice compared to WT littermates. (6) Most importantly, overexpression of miR29a and HDAC4 RNAi significantly attenuated the activated HSC migration and proliferation. Knockdown of miR-29a with an antisense inhibitor promoted HDAC4 function and restored HSC migration and accelerated proliferation. These phenomena rationalize our hypotheses to focus on the molecular events underlying miR-29a protection against BDL or hydrophobic bile acid-mediated HSC activation.

Obstructive jaundice has been shown to be associated with the transcriptional activation of pro-inflammatory cytokines. Previously, we have demonstrated an increase in MCP-1 expression in cholestatic liver and in isolated HSCs [17]. MCP-1 is one of the most significant chemokines regulating the recruitment and maintenance of inflammatory infiltrates during liver injury [28]. Activated HSCs and biliary epithelial cells are responsible for MCP-1 production and HSC recruitment and activation in chronic liver disease [28, 29]. Herein, we showed that there was lower MCP-1 expression in isolated HSCs from miR-29aTg mice than in WT littermates.

Acetylation of lysine residues modulates protein-histone and histone-DNA interactions, and thereby regulates many cellular processes [30]. HDAC inhibitors have been extensively studied in experimental models of cancer, where their inhibition of deacetylation has been proven to regulate cell survival, proliferation, differentiation and apoptosis [31]. Currently, numerous HDAC inhibitors, including trichostatin A, valproic acid, and Largazole, have been identified as potent inhibitors of HSC activation both *in vitro* and *in vivo* that could reduce inflammatory activity and liver fibrosis [31–33]. In addition, inhibition of HDAC activity leads to a strong reduction of HSC activation markers, α-SMA, lysyl oxidase and collagens, as well as an inhibition of cell proliferation through the induction of miR-29 expression [10]. Moreover, our group has demonstrated that HDAC4 interference increases the acetylation status of H3K9, which is enriched in the miR-29a proximal promoter, and reduces miR-29a transcription in high glucose-stressed podocytes [12]. In contrast, overexpression of miR-29a promotes nephrin acetylation that ameliorates hyperglycemia-induced podocyte dysfunction through inhibition of HDAC4 signaling transduction [12]. In addition, we demonstrated that miR-29a signaling protected against glucocorticoid-induced osteoporosis and improved osteoblast differentiation and mineral acquisition [13] through reduced HDAC4 signaling [34].

**Fig 10:**
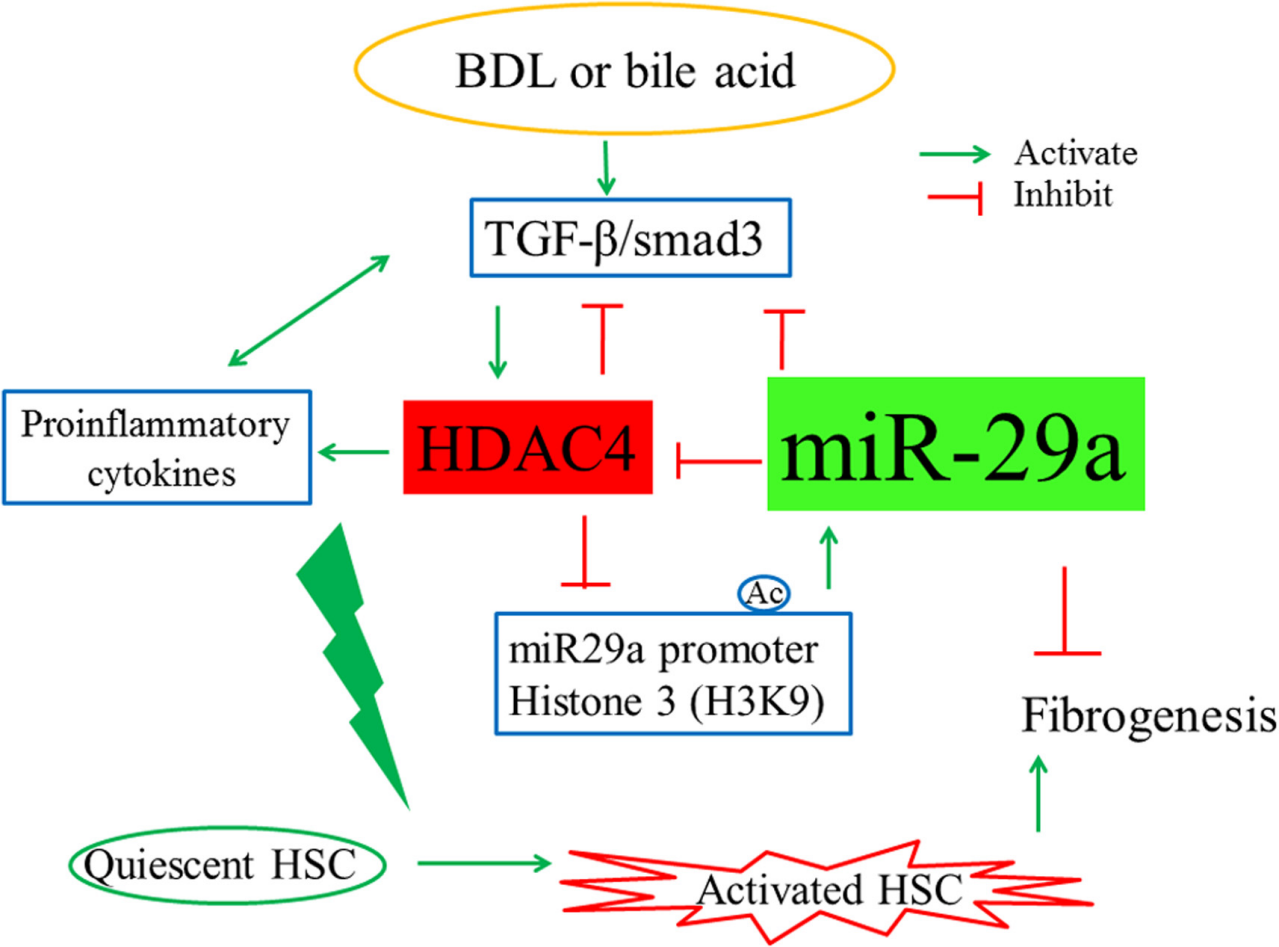
Proposed model of miR-29a signaling protection in liver fibrosis and HSC activation. MiR-29a is an important regulator of the profibrogenic phenotype of HSCs. By suppressing HDAC4 action, miR-29a increases H3K9 acetylation and suppresses Smad3 phosphorylation; therefore inhibiting the activation of HSCs.

In addition, miR-29 is also a major regulator of genes associated with pulmonary fibrosis [35], renal fibrosis [26], as well as myocardial infarction [36] and aneurysm formation [37]. It seems that miR-29 is a key player in fibrogenesis. We first demonstrated that overexpression of miR-29a in cholestatic mice significantly inhibited collagen-1α1 and collagen-4α1 protein expression in miR-29aTg mice with cholestasis compared with the WT littermates [14]. Previously, we showed that hepatic overexpression of miR-29 leads to inhibition of hepatocellular apoptosis and to reduction of acute liver damage [14]. Therefore, stellate cell activation might not be only attenuated by stellate cell specific direct effects by miR-29a overexpression, but also by the indirect influence of less hepatocyte injury. In particular, inhibition of apoptosis of hepatocytes upon miR-29aTg overexpression is suggested to result in diminution of stellate cell activation due to the reduction of the inflammatory reaction in response to fewer hepatocellular apoptotic bodies [38, 39]. Stimulation of HSCs by TGF-β is a crucial event in liver fibrogenesis due to its impact on myofibroblastic transition and ECM induction. TGF-β secretion by hepatocytes, Kupffer cells, and sinusoidal endothelial cells causes HSC to activate, transdifferentiate, and secrete ECM [40]. Recently, Roderburg *et al*. reported that TGF-β1- mediated downregulation of miR-29 in HSCs [5], a finding supported by Bandyopadhyay *et al*. [6]. In a recent study of renal fibrosis, it was demonstrated that Smad3 mediated TGF-β1-induced the downregulation of miR-29 by binding to the promoter of miR-29 [26]. Furthermore, miR-29 acted as a downstream inhibitor and therapeutic miR for TGF-β1/Smad3-mediated renal fibrosis. Moreover, miR-29 can inhibit the TGF-β1-mediated upregulation of HDAC4 via the inhibition of Smad3 expression in the regulation of myogenic differentiation [19]. It is also consistent with our findings that overexpression of miR-29a could downregulate p-Smad3 and HDAC4 expression *in vivo* and *in vitro*. Thus, miR-29a is an important regulator of the profibrogenic phenotype of HSCs and plays as an important role of the cross-talk between HDAC4 and TGF-β1 signaling (Fig 10). By suppressing HDAC4 action, miR-29a restores the acetylation status of H3K9. It also can suppress Smad3 phosphorylation and thereafter inhibits the activation of HSCs. Moreover, in our previous study, overexpression of miR-29a significantly reduced the expression of pro-apoptotic proteins, inhibition of NF-κB activation and enhanced phospho-AKT protein expression, thereby leading to a decrease in hepatocellular apoptosis in cholestasis [14]. Taken together, miR-29a is an important regulator in the maintenance of HSC ultrastructure integrity and liver homeostasis.

Inhibition of the fibrogenic, proliferative, and migratory effects of HSCs is an emerging experimental therapy for the prevention and regression of hepatic fibrosis. This study highlights an emerging view of an epigenetic mechanism that the activation of HSCs by miR-29a signaling may modulate their profibrogenic phenotype, thus supporting the use of miR-29a agonists as a potential therapy to treat liver fibrosis in the future.

## Supporting Information

S1 Fig Oil red O staining and α-smooth muscle actin (α-SMA) in the hepatic stellate cell (HSC). After 1 day in culture, the HSCs have a quiescent phenotype (A) and they develop an activated phenotype that reveal the unique appearance of star-shaped and lose of lipid droplets after 8 days of culture (B). A characteristic hallmark of activated HSCs is the expression of α-SMA (C, right) compared to quiescent phenotype (C, left).
(TIF)
