# Supplementary figures and images for: Activation of Mir-29a in Activated Hepatic Stellate Cells Modulates Its Profibrogenic Phenotype through Inhibition of Histone Deacetylases 4
Source: PLoS One. 2015 Aug 25;10(8):e0136453. doi: 10.1371/journal.pone.0136453 (PMC4549141; doi:10.1371/journal.pone.0136453)

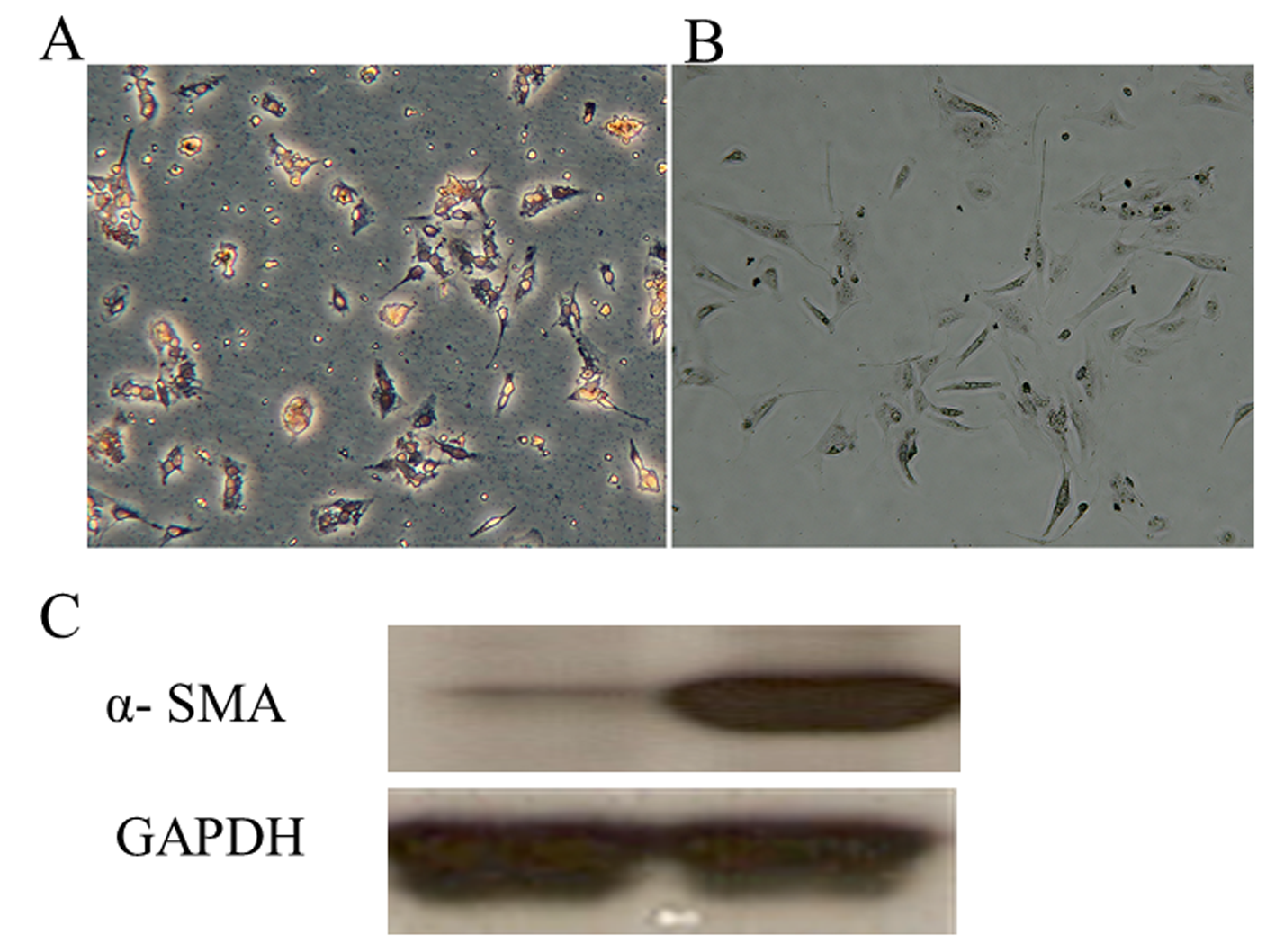

Supplement: S1 Fig — After 1 day in culture, the HSCs have a quiescent phenotype (A) and they develop an activated phenotype that reveal the unique appearance of star-shaped and lose of lipid droplets after 8 days of culture (B). A characteristic hallmark of activated HSCs is the expression of α-SMA (C, right) compared to quiescent phenotype (C, left). (TIF) [file pone.0136453.s001.tif]
